# Cancer wars: natural products strike back

**DOI:** 10.3389/fchem.2014.00020

**Published:** 2014-05-01

**Authors:** Christine Basmadjian, Qian Zhao, Embarek Bentouhami, Amel Djehal, Canan G. Nebigil, Roger A. Johnson, Maria Serova, Armand de Gramont, Sandrine Faivre, Eric Raymond, Laurent G. Désaubry

**Affiliations:** ^1^Therapeutic Innovation Laboratory, UMR7200, CNRS/University of StrasbourgIllkirch, France; ^2^AAREC Filia ResearchClichy, France; ^3^L.C.I.M.N Laboratory, Department of Process Engineering, Faculty of Technology, University Ferhat AbbasSétif, Algeria; ^4^Biotechnology and Cell Signaling Laboratory, UMR 7242, CNRS/ University of StrasbourgIllkirch, France; ^5^Department of Physiology and Biophysics, State University of New YorkStony Brook, NY, USA; ^6^Department of Medical Oncology, Beaujon University Hospital, INSERM U728/AP-HPClichy, France

**Keywords:** natural products, cancer, drug discovery, pharmacognosy, molecular targets, privileged structures

## Abstract

Natural products have historically been a mainstay source of anticancer drugs, but in the 90's they fell out of favor in pharmaceutical companies with the emergence of targeted therapies, which rely on antibodies or small synthetic molecules identified by high throughput screening. Although targeted therapies greatly improved the treatment of a few cancers, the benefit has remained disappointing for many solid tumors, which revitalized the interest in natural products. With the approval of rapamycin in 2007, 12 novel natural product derivatives have been brought to market. The present review describes the discovery and development of these new anticancer drugs and highlights the peculiarities of natural product and new trends in this exciting field of drug discovery.

## Introduction

Recent analyses of tooth plaques showed that ~50,000 years ago Neanderthals already used medicinal plants to treat their ailments (Hardy et al., 2012). Currently, more than half of humanity does not have access to modern medicine and relies on traditional treatments (Cordell and Colvard, 2012). A recent analysis of the strategies used in the discovery of new medicines showed that 36% of the first-in-class small-molecules approved by U.S. Food and Drug Administration (FDA) between 1999 and 2008 were natural products or natural products derivatives (Swinney and Anthony, 2011).

Natural products are small-molecule secondary metabolites that contribute to organism survival. These substances display considerable structural diversity and “privileged scaffolds,” i.e., molecular architectures that are tailored to protein binding, as first coined by Evans in the late 1980s (Evans et al., 1988). Indeed natural products have evolved to bind biological targets and elicit biological effects as chemical weapons or to convey information from one organism to another. Steroid derivatives are often not considered as natural products because their design is not based on a research in pharmacognosy, however we subjectively decided to include them here due to their importance in drug discovery.

The synthesis of aspirin by Charles Gerhard at Strasbourg faculty of pharmacy in 1853 paved the road for the medicinal chemistry of natural products (Gerhardt, 1853). In 1964, actinomycin became the first natural product approved for an indication in oncology. Other natural products based medicines such as anthracyclines, *vinca* alkaloids, epipodophyllotoxin lignans, camptothecin derivatives, and taxoids that were launched before 1997, are still an essential part of the armament for treating cancers.

From 1997 to 2007 no new natural product was approved for the treatment of cancer (Bailly, 2009). With the imminent achievement of the genome project, the head of a pharmaceutical company declared that natural products were outdated. Their development was greatly reduced and many big pharmaceutical companies closed their departments of natural product chemistry (Bailly, 2009). The future was targeted therapies, which uses fully synthetic molecules or antibodies to target specific proteins in tumor growth and progression. In some forms of leukemia, gastrointestinal, prostate or breast cancers, targeted therapies greatly delayed tumor progression, and/or improved the life expectancy of the patients. Some tumors with specific oncogenic addictions (for example fusion proteins leading to ALK expression in lung cancer or Bcr-Abl in chronic myeloid leukemia, KIT expression or mutations in GIST or EGFR mutation in lung cancer, HER2 amplification in breast cancer or MET overexpression in liver tumors) greatly benefited from targeted agents. However, the vast majority of common tumors were found to be not dependent of a single “targetable” oncogenic activation. For instance altogether ALK activations and EGFR mutations account for less than 10% of lung adenocarcinoma and while those targeted agents are more efficient than chemotherapy in oncogenic tumors, antitumor effects are limited to few months. Importantly, most tumors were shown to activate multiple signaling pathway redundancies and adaptive mechanisms that either render tumors primarily resistant to targeted drugs or facilitate acquired resistance to cell signaling inhibition after only few months of treatments. As a result, the expected progression-free survival benefit from targeted therapy is often less than 6-months. For those later forming complex but rather frequent tumors, chemotherapy alone remains the cornerstone of treatment with some limited add-on benefits by use of monoclonal antibodies in a limited proportion of patients. Combinations of several targeted agents have also been proposed to counteract potential adaptive mechanisms although one should notice that combining targeted agent together was more often associated with unacceptable toxicity than great clinical synergy. Then there is the additional influence of cost-to-benefit concerns. The financial cost of such targeted therapies, to patients or health insurance entities, can be considered enormous, e.g., thousands to tens of thousands of euros per day of extended life. However, the net financial benefit to pharmaceutical companies of those agents that are given only for few months (or years) in only a small proportion of patients in niche indications may lead to restricted investment by pharmaceutical industries; blockbuster indications usually provide higher revenues.

These drawbacks are at the origin of the re-emergence of natural products in oncology. Since 2007, with the approval of rapamycin and derivatives of it, 12 natural product derivatives have been approved for the treatment of cancers (Table 1).

**Table 1:** **Novel anticancer medicines based on natural products**.

**Name (trade name), structure**	**Year of approval, company**	**Therapeutic indication, mode of action**
Temsirolimus (Torisel®): R=R^1^	2007, Wyeth	Treatment of renal cell carcinoma (RCC), inhibition of mTOR
Everolimus (Afinitor®), R=R^2^		
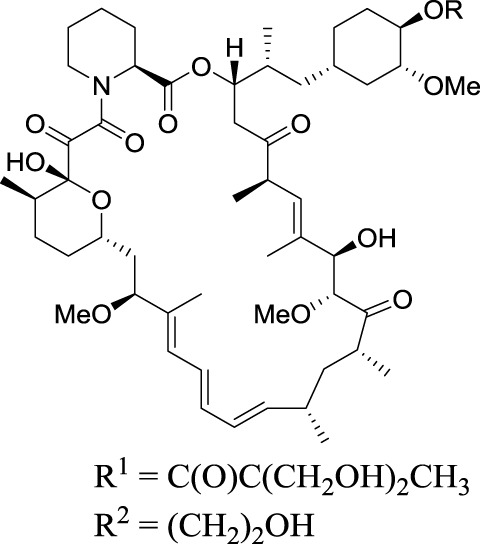	2009, Novartis	Treatment of advanced kidney cancer, inhibition of mTOR
Ixabepilone (Ixempra®)	2007, Bristol-Myers Squibb	Treatment of aggressive metastatic or locally advanced breast cancer no longer responding to currently available chemotherapies, stabilization of microtubules
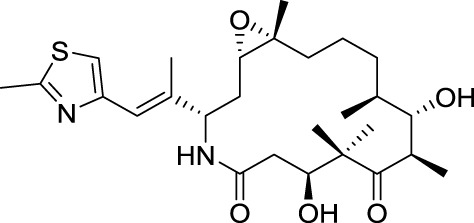		
Vinflunine (Javlor®)	2009, Pierre Fabre	Treatment of bladder cancer, inhibition of tubulin polymerization
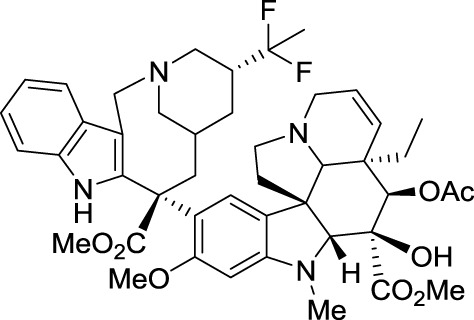		
Romidepsin (Istodax®)	2009, Celgene	Treatment of cutaneous T-cell lymphoma (CTCL), inhibition of the isoforms 1 and 2 of histone deacetylases
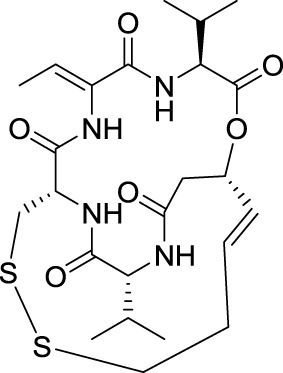		
Trabectedin = ecteinascidin 743 (Yondelis®)	2009, Zeltia and Johnson and Johnson	Treatment of advanced soft tissue sarcoma and ovarian cancer, induction of DNA damage
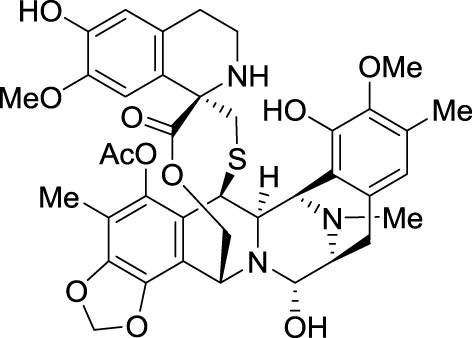		
Cabazitaxel (Jevtana®)	2010, Sanofi-Aventis	Treatment of hormone-refractory metastatic prostate cancer, microtubule stabilization
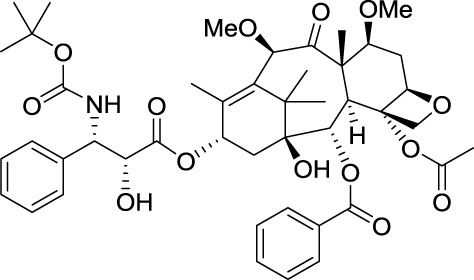		
Abiraterone acetate (Zytiga®)	2011, Janssen	Treatment of castration-resistant prostate cancer, inhibition of 17 α-hydroxylase/C17, 20 lyase (CYP17A1)
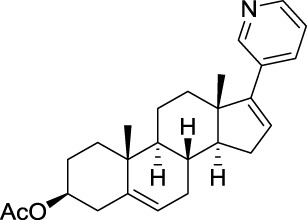		
Eribulin mesylate (Halaven®)	2011, Eisai Co.	Treatment of metastatic breast cancer, inhibition of microtubule dynamics
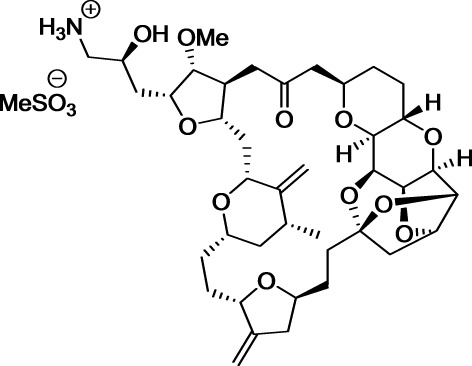		
Homoharringtonine, Omacetaxine mepesuccinate (Synribo®)	2012, Teva	Chronic myelogenous leukemia (CML), inhibition of protein synthesis
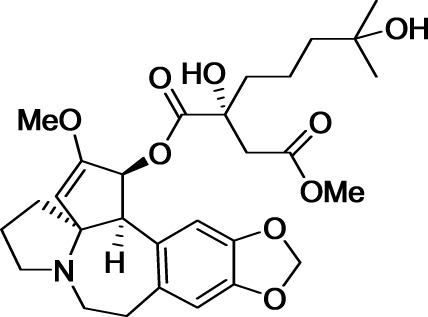		
Carfilzomib (Kyprolis®)	2012, Onyx	Treatment of multiple myeloma, inhibition of proteasome
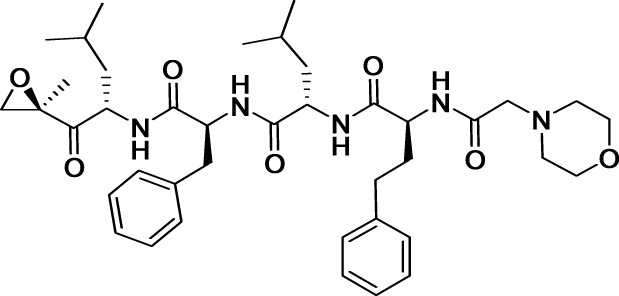		
Ingenol mebutate (Picato®)	2012, LEO Pharma	Actinic keratosis, activation of PKCδ
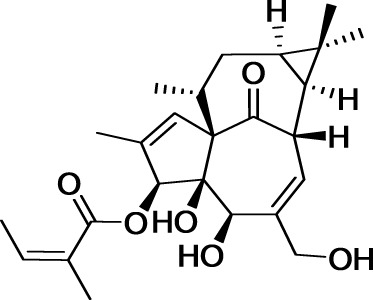		

Recently Stuart Schreiber, Paul Clemons and coworkers at the Broad Institute in Boston performed a bioinformatics analysis of natural product targets and demonstrated that natural products statically tend to target proteins with a high number of protein–protein interactions that are particularly essential to an organism (Dančík et al., 2010). This observation is consistent with the common role played by natural products as chemical weapons against predators or competitors.

Henkel et al. at Bayer AG in Germany offered a statistical analysis of the structural differences between natural products and fully synthetic drugs (Henkel et al., 1999). Compared with fully synthetic drugs, natural product tend to have more chiral centers, more oxygen atoms, less nitrogen atoms, and more varied ring systems. Complementary analyses of structural features of natural products have been reviewed (Lee and Schneider, 2001; Ortholand and Ganesan, 2004; Ganesan, 2008; Grabowski et al., 2008). A consequence of this structural complexity is that natural products tend to be more selective toward their targets than fully synthetic drugs, and consequently rarely display off-target—induced iatrogenicity.

Moreover, complex natural products tend to act through only one class of molecular target, even though there are some exceptions. Indeed, taxanes are known to target β-tubulin and interfere with microtubule dynamics; however they also bind to Bcl-2 to block its anti-apoptotic activity. Both β-tubulin and Bcl-2 interact with the orphan nuclear receptor Nur77 (NGFI-B, TR3, NR4A1). Ferlini et al. showed that in fact taxanes mimic the domain of Nur77 involved in the interaction with β-tubulin and Bcl-2 (Ferlini et al., 2009). Another example concerns flavaglines, an emerging family of natural compounds found in medicinal plants of South-East Asia, which display potent anticancer effects through their direct effects on the scaffold proteins prohibitins and the initiation factor of translation eIF4a (Basmadjian et al., 2013; Thuaud et al., 2013).

**Figure 1:**
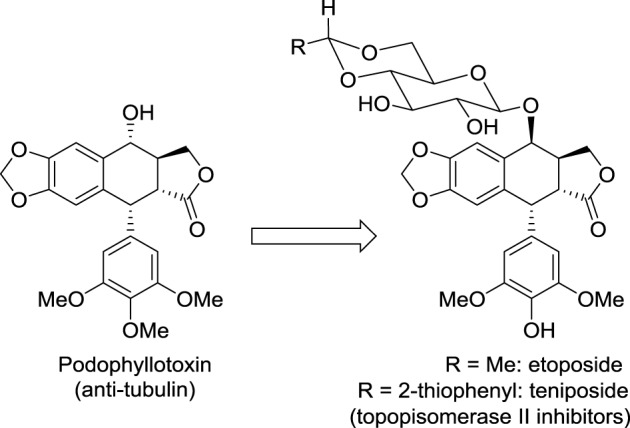
**Structures of podophyllotoxin, etoposide, and teniposide**.

Modifying the structure of a drug may change the nature of its molecular target. A striking example concerns the not so rational development of the anticancer medicines etoposide and teniposide (Figure 1). Considering that cardiac glycosides display enhanced pharmacological properties compared to the cognate aglycone, Sandoz scientists hypothesized that conjugating podophyllotoxin to a glucose moiety could improve the activity of this cytotoxic agent that binds tubulin and inhibits assembly of the mitotic spindle. Fortunately, this glycoconjugate named etoposide displayed a promising anticancer activity with reduced adverse effects compared with podophyllotoxin. Surprisingly, etoposide did not affect tubulin polymerization but inhibited another very important target in oncology: DNA topoisomerase II. This story illustrates well the importance in drug discovery of serendipity, which was likened to “looking for a needle in a haystack and discovering the farmer's daughter” by Professor Pierre Potier, inventor of the anticancer drug taxotere (Zard, 2012).

Another non-rational issue regarding the SAR of derivatives of natural compounds concerns the relationship between the chemical structure of a drug and its therapeutic indication. Indeed, transforming the structure of a drug may modify the nature of the targeted cancer. This is well established for *vinca* alkaloids for instance (Table 2). If we could understand the influence of the molecular structure of a drug with its optimal therapeutic indication, then we might be able to adapt known medicines to treat cancers that are reluctant to current therapies.

**Table 2:** **Structures and therapeutic indications of *vinca* alkaloids**.

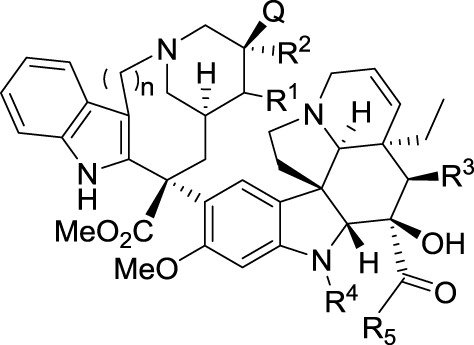								
**Name**	***n***	**Q**	**R^1^**	**R^2^**	**R^3^**	**R^4^**	**R^5^**	**Therapeutic indication**
Vinblastine	2	OH	H	Et	OAc	Me	OMe	Lymphomas, germ cell tumors, breast, head and neck cancer and testicular cancers
Vinorelbine	1	Q=R^1^ = ∅ (alkene)		Et	OAc	Me	OMe	Osteosarcoma, breast, and non-small cell lung cancers
Vincristine	2	OH	H	Et	OAc	CHO	OMe	Acute lymphoblastic leukemia, rhabdomyosarcoma, neuroblastoma, lymphomas, and nephroblastoma
Vindesine	2	OH	H	Et	OH	Me	NH_2_	Melanoma, lung, breast and uterine cancers, leukemia and lymphoma
Vinflunine	1	H	H	CF_2_Me	OAc	Me	OMe	Bladder cancer

In spite of the major achievements in systems biology and translational medicine over the last decade, there is still, at best, a presumptive relationship between the efficacy of a drug in preclinical assays and the likelihood of its value in clinic.

## Rapalogues: Temsirolimus® and Everolimus®

In 1975, researchers at Ayerst Laboratories (Canada) reported the isolation of rapamycin as a secondary metabolite from a strain of *Streptomyces hygroscopicus* based on its antifungal activity (Sehgal et al., 1975; Vezina et al., 1975). Its name comes from Rapa Nui (Easter Island) where its producer strain had been collected from a soil sample. Its richly adorned macrocyclic structure was fully elucidated a few years later (Swindells et al., 1978; Findlay and Radics, 1980; McAlpine et al., 1991). Rapamycin did not attract so much attention until the discovery in 1987 of the structurally related immunosuppressant FK506 (Kino et al., 1987a,b). Rapamycin was eventually developed without further structural modifications as the oral immunosuppressant drug sirolimus. It was approved for prevention of rejection in organ transplantation in 1999 (Calne et al., 1989; Kahan et al., 1991; Watson et al., 1999; Calne, 2003).

Determining the mode of action of rapamycin unraveled one of the most important signaling pathways in cell biology, which illustrates another important aspect of the pharmacology of natural products. Indeed a common caveat of developing an original natural product toward clinical application is the requirement to identify its molecular target and understand its mode of action (Krysiak and Breinbauer, 2012). However, when the target is identified, it may lead to major breakthroughs in cell biology (Pucheault, 2008). Gratefully, current technologies render this task increasingly easier (Ares et al., 2013).

In 1991, Michael Hall et al. identified the molecular target of rapamycin in a gene complementation assay in yeast and named it TOR for “Target Of Rapamycin” (Hietman et al., 1991). Three years later, Stuart Shreiber et al. identified its mammalian homolog referred to today as the kinase mTOR (mammalian TOR) (Brown et al., 1994). The mode of action of rapamycin is unique: it binds to two proteins at the same time, mTOR and the immunophilin FKBP-12, to form a ternary complex devoid of any kinase activity. mTOR plays a central role integrating signals from growth factors, nutrients, stress, and hormones to regulate metabolism, proliferation, cell growth, and apoptosis. However, the exact mechanisms of action of rapamycin derivatives, called rapalogues, remain only moderately understood. Some recent evidence indicates that rapalogues may primarily display their anticancer effects through an inhibition of angiogenesis in patients (Faivre and Raymond, 2008). This hypothesis would explain why rapologues are particularly effective in hypervascularized tumors.

Currently, two rapalogues, temsirolimus, and everolimus, have been developed for the treatment of renal, breast, and pancreas cancers, astrocytoma, and mantle cell lymphoma. These drugs differ in their formulation, application, and dosing schemes, thereby yielding varying bioavailabilities. They are all prepared by semi-synthesis.

## Ixabepilone (Ixempra®)

**Figure 2:**
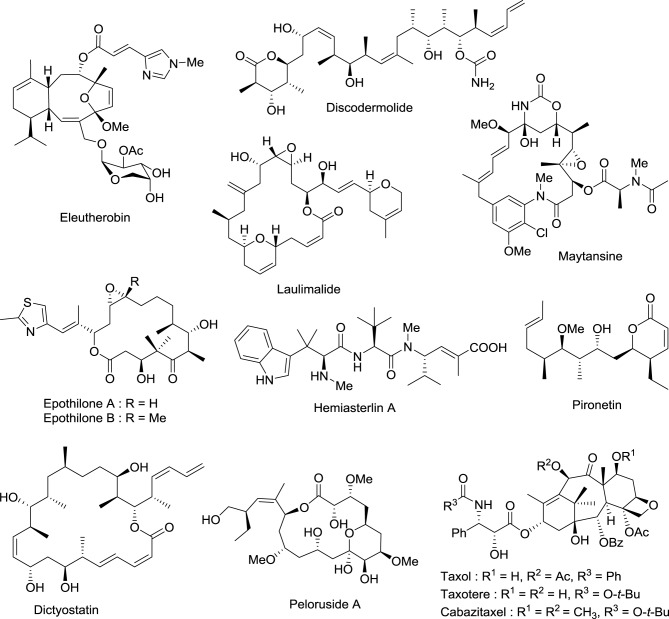
**Chemical structures of representative natural compounds that stabilize microtubule assemblies**.

Drugs that target microtubules, such as taxoids and *vinca* alkaloids, continue to represent an important class of chemotherapeutic agents (Jordan and Wilson, 2004). Over the last two decades other classes of naturally occurring nontaxoid compounds, the epothilones (Gerth et al., 1996; Höfle et al., 1996), discodermolides (Gunasekera et al., 1990), eleutherobins (Lindel et al., 1997), and laulimalides (Mooberry et al., 1999) that stabilize microtubule assemblies similarly to taxol, have been identified (Figure 2). Based upon extensive structure-activity data, a common pharmacophore for these different classes of compounds has been proposed (Ojima et al., 1999).

Not only is epothilone B more cytotoxic than taxol, but it is also much less sensitive toward the development of multidrug-resistance, a major concern in the clinic (Horwitz, 1994; Bollag et al., 1995; Kirikae et al., 1996). This impressive pharmacological profile coupled with the challenge of its total synthesis has attracted the attention of some of the most well-known organic chemists in the world, including Samuel Danishefsky (Balog et al., 1996; Su et al., 1997), followed by Nicolaou (Nicolaou et al., 1997; Yang et al., 1997), Schinzer (Schinzer et al., 1997), Mulzer (Mulzer et al., 2000) and Carreira (Bode and Carreira, 2001).

Early investigations indicated that natural epothilones display poor metabolic stability and unfavorable pharmacokinetic properties (Lee et al., 2000). Several synthetic and semi-synthetic analogs were then examined and evaluated in preclinical studies. Eventually, isosteric replacement of the lactone by a lactam afforded ixabepilone (also known as azaepothilone B) (Lee et al., 2008). Not only is this drug not susceptible to hydrolysis by esterases, conferring metabolic stability, but it also displays improved water solubility, which greatly alleviate galenic problems associated with hypersensitivity reaction in patients.

In 2007, the FDA (but not its European equivalent, European Medicines Agency or EMA) approved ixabepilone for the treatment of aggressive metastatic or locally advanced breast cancer no longer responding to currently available chemotherapies.

## Vinflunine (Javlor®)

*Vinca* alkaloids were the first chemotherapeutic agents that target microtubules. The first member of this class, vinblastine, was isolated in 1958 (Noble et al., 1959). Latter, some derivatives, vincristine, vinorelbine, and finally avelbine, were developed to treat hematological and solid malignancies in both adult and pediatric patients (Table 1). *Vinca* alkaloids block the polymerization of tubulin molecules into microtubules to prevent the formation of the mitotic spindle.

**Scheme 1:**
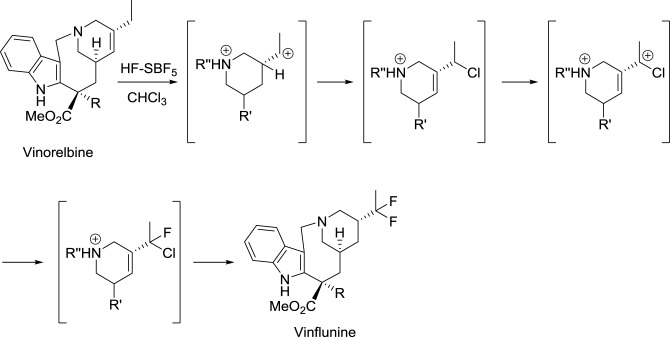
**Synthesis of vinflunine from vinorelbine (Fahy et al., 1997)**.

In the course of their study on the reactivity of functionalized molecules in superacid media, Jacquesy and collaborators found that the treatment of vinorelbine with a combination of HF and SbF_5_ gave a difluoro analog, later called vinflunine (Scheme 1) (Fahy et al., 1997). Importantly, this new compound displayed an enhanced bioavailability compared to other *vinca* alkaloids. Indeed, its terminal half-life was calculated to be about 40 h and the terminal half-life for its active metabolite (4-*O*-deacetylvinflunine) was reported to be 4–6 days in several phase I trials (Bennouna et al., 2003, 2005; Johnson et al., 2006).

Resistance to vinflunine develops more slowly than with other *vinca* alkaloids. In addition, vinflunine *in vitro* neurotoxicity is lower than that of vincristine or vinorelbine (Etiévant et al., 1998, 2001; Estève et al., 2006). Further development by Pierre Fabre and Bristol Myers Squibb laboratories ended with the approval of vinflunine for the treatment of bladder cancer by the European Medicines Agency (EMA) in 2009.

## Romidepsin (Istodax®)

The cyclic depsi-pentapeptide romidepsin, also called FR901228, FK228, or NSC 630176, was isolated and identified by Ueda and colleagues at Fujisawa Pharmaceutical in Japan through a screening program of fermentation products able to revert the transformed morphology of a Ha-ras NIH3T3 cells to normal (Ueda et al., 1994). Indeed Ha-ras is an oncogene involved in tumorigenesis and consequently represents an important target in oncology. Importantly, romidespsin displayed potent antitumor activities against A549 and MCF-7 tumors in xenografted mice. These results attracted the attention of NCI scientists who continued to explore its anticancer properties under a Cooperative Research and Development Agreement with Fujisawa Corporation (now Astellas).

When romidepsin was discovered, histone deacetylases (HDAC) were emerging as important targets for the treatment of cancer (Thaler and Mercurio, 2014). Screening of microbial metabolites for their effects on transcription showed that romidepsin behaves similarly to trichostatin A, a known HDAC inhibitor (Nakajima et al., 1998). Romidepsin acts as a prodrug, which is reduced in cells to its active form by glutathione, yielding a monocyclic dithiol that preferentially inhibits the isoforms HDAC1 and HDAC2 (Furumai et al., 2002).

In 2002, when it became established that romidepsin holds a promising therapeutic potential, Fujisawa Corporation began clinical trials. Romidepsin was licensed to Gloucester Pharmaceuticals in 2004 (latter acquired by Celgene Co) and was approved by the FDA in 2009 for the treatment of cutaneous T-cell lymphoma. The preclinical and clinical development has been described in an excellent review (Vandermolen et al., 2011).

## Ecteinascidin 743 = trabectedin (Yondelis®)

In 1969, unidentified alkaloids from the Caribbean tunicate *Ecteinascidia turbinate* were shown to display some anticancer activities, but the structure of these complex alkaloids could not be determined because of their natural scarcity and the limitation of analytical chemistry at that time (Sigel et al., 1970). In 1990, Rinehart et al. from the University of Illinois at Urbana-Champaign elucidated the structure and reported the cytotoxicity of these tetrahydroisoquinoline alkaloids, the ecteinascidins (Rinehart et al., 1990). These compounds displayed greater *in vitro* and *in vivo* antitumor activity than those reported for the structurally related microbial metabolites saframycins and safracins.

**Scheme 2:**
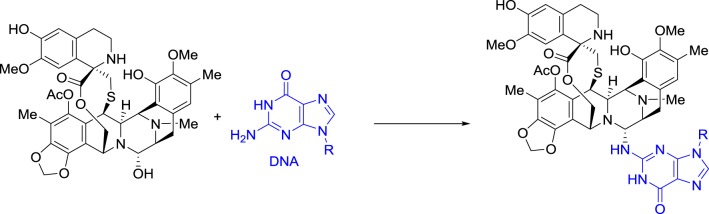
**DNA Alkylation by ecteinascidin 743**.

Ecteinascidin 743, also called trabectedin and ET-743, was then selected for preclinical development based on its exceptional *in vitro* cytotoxicity. Pommier et al. at NCI demonstrated that this drug binds in the minor groove of DNA and alkylates the exocyclic amino group at position 2 of guanine in GC-rich regions (Scheme 2) (Pommier et al., 1996).

Ecteinascidin 743 was shown to block the DNA excision repair system (Takebayashi et al., 2001; Zewail-Foote et al., 2001), to cross-link DNA with topoisomerase I (Martinez et al., 1999; Takebayashi et al., 1999; Zewail-Foote and Hurley, 1999), and also to inhibit the binding of DNA to some transcription factors (Bonfanti et al., 1999; Jin et al., 2000; Minuzzo et al., 2000). However, the cascade of events that links DNA damage to the resulting antitumor activity is far from being understood (D'Incalci and Galmarini, 2010).

When ecteinascidin 743 was licensed to PharmaMar; this company launched a very challenging program of aquaculture to produce sufficient quantities of tunicate biomass to feed clinical trials program. After several years of intensive development, the cumulative total biomass reached some 250 metric tons. However, isolation of ecteinascidin 743 required complex and costly steps of purification with final yields less than 1 g/ton (Cuevas and Francesch, 2009). Several total syntheses have been reported, but they cannot be translated into industrial production (Corey et al., 1996; Endo et al., 2002; Chen et al., 2006; Zheng et al., 2006; Fishlock and Williams, 2008; Imai et al., 2012; Kawagishi et al., 2013). Eventually, this supply problem was solved by use of a complex semi-synthesis from cyanosafracin B, which is available in kilogram quantities by fermentation (Cuevas et al., 2000; Menchaca et al., 2003).

Preclinical studies did not reveal that soft tissue sarcoma is more sensitive to ecteinascidin 743 than other solid tumors. This response was unveiled first during phase I clinical trials and confirmed in phase II (Taamma et al., 2001; Villalona-Calero et al., 2002; D'Incalci and Jimeno, 2003). This drug was approved under the name of Yondelis in 2007 in the European Union for the treatment of patients with advanced soft tissue sarcoma. This compound was the first anticancer medicine of marine origin to be approved. It was followed by eribulin (vide infra), validating the concept that marine natural products should be considered important contenders in drug discovery.

## Cabazitaxel (Jevtana®)

The taxane anticancer drug cabazitaxel is a semi-synthetic derivative of the natural taxoid 10-deacetylbaccatin III. It was approved in 2010 by the FDA, in combination with prednisone, for the treatment of patients with hormone-refractory metastatic prostate cancer who had already been administered a treatment containing the taxane docetaxel (Galsky et al., 2010). In 2013, Vrignaud et al. showed that *in vitro*, cabazitaxel stabilized microtubules as effectively as docetaxel but was also 10 times more potent than docetaxel in chemotherapy-resistant tumor cells. They also noted that cabazitaxel was active in docetaxal-resistant tumors (Vrignaud et al., 2013). In addition, cabazitaxel penetrates the blood-brain barrier. Cabazitaxel was approved 20 years after taxol, illustrating that there is still room to improve well established anticancer medicines.

## Abiraterone acetate (Zytiga®)

Abiraterone acetate is an oral inhibitor of androgen synthesis used since 2011 for the treatment of castration-resistant prostate cancer. Previous treatments of prostatic cancers prevented androgen production by the testes, but not by the adrenals. Abiraterone acetate is rapidly hydrolyzed *in vivo* to abiraterone, which is a selective, irreversible inhibitor of cytochrome P450 17α (CYP17), an enzyme that catalyzes the conversion of pregnenolone and progesterone into DHEA or androstenedione, two precursors of testosterone. This drug was originally designed and synthesized by Jarman et al. at the Institute of Cancer Research in Sutton (UK) based on the hypothesis that the nitrogen lone pair of a pyridyl moiety linked to the steroid skeleton would coordinate with the iron atom of the heme cofactor in the active site of CYP17 (Potter et al., 1995; Jarman et al., 1998).

The inhibition of CYP17 by abiraterone acetate blocks androgen biosynthesis and significantly improves the therapy of castration-resistant prostate cancer, which remains a challenge to treat (Rehman and Rosenberg, 2012). Unfortunately, this CYP17 inhibition also decreases glucocorticoid and increases mineralocorticoid production, which results in the main source of adverse effects.

**Figure 3:**
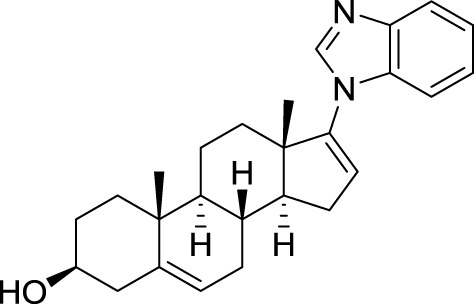
**Structure of galeterone**.

Since the invention of abiraterone, different steroids bearing a heteroaromatic substituent on the D ring continued to be developed as CYP17 inhibitors. Among those, galeterone (TOK-001 or VN/124-1) recently entered clinical trials for the treatment of chemotherapy-naive, castration-resistant prostate cancer (Figure 3) (Vasaitis and Njar, 2010). Interestingly, this drug not only inhibits CYP17, but is also an androgen receptor antagonist (Handratta et al., 2005).

## Eribulin mesylate (Halaven®)

In 1985, Uemura et al. isolated and identified norhalichondrin A from the marine sponge *Halichondria okadai* based on its potent *in vitro* toxicity (Uemura et al., 1985). Related polyether macrolides, including halichondrin B (Hirata and Uemura, 1986) were identified in the following years (Qi and Ma, 2011). Tests with NCI's 60-cell line screen suggested that halichondrin B affects tubulin polymerization. Further studies showed that this drug displays subtle differences in mechanism of action from those of other known antimitotics targeting tubulin. Although halichondrin B displayed promising activity, its preclinical investigation has been hampered by its scarcity from natural sources.

Due to its complexity, the total synthesis of halichondrin B was considered as an attractive objective by Kishi et al. at Harvard University. This team achieved this formidable challenge in 1992 (Aicher et al., 1992). Further collaborative studies from this group and Eisai Co. ultimately led to the development of the simplified and pharmaceutically improved analog eribulin (Jackson et al., 2009). Although it is less complex than natural halichondrins, eribulin contains 19 stereogenic centers, two exocyclic olefins, seven polyoxygenated pyrans and tetrahydrofurans, a 22-membered macrolactone ring, and a 36 carbon backbone. With its 35 steps, eribulin synthesis extended the limit of feasibility for industrial production. Indeed, eribulin is the single most complex molecule synthesized at an industrial scale and represents an awe-inspiring testimony to the current power of organic synthesis. Eribulin was approved by FDA in 2010 to treat patients with metastatic breast cancer who have received at least two prior chemotherapy regimens for late-stage disease.

## Homoharringtonine = omacetaxine mepesuccinate (Ceflatonin®)

**Figure 4:**
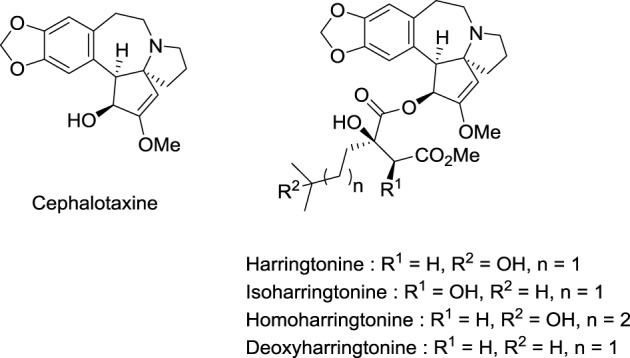
**Structures of cytotoxic *Cephalotaxus* alkaloids**.

Toxic seeds of the conifer *Cephalotaxus harringtonia K. Koch* var*harringtonia* belong to the traditional Chinese pharmacopeia. In observance with Mao Tse-tung's judgment that Chinese medicine and pharmacology represent a national treasure that needs to be valorized, Chinese investigators established that the total alkaloids from C*ephalotaxus fortunei Hook.f* possesses antitumor activity in preliminary clinical trials (Group, 1976). In the same period, National Cancer Institute (NCI) scientists found that *Cephalotaxus harringtonia* seed extracts displayed significant *in vivo* activity against L-1210 and P-388 leukemia tumors in mice. Powell et al. from the U.S. Department of Agriculture isolated and identified the structure of cytotoxic *Cephalotaxus* alkaloids: harringtonine, isoharringtonine, homoharringtonine, and deoxyharringtonine (Powell et al., 1970) (Figure 4). These compounds are esters of cephalotaxine, an inactive alkaloid first isolated by Paudler et al. in 1963 at Ohio University (Paudler et al., 1963). Homoharringtonine was found to be the most effective in prolonging survival of P388 leukemic mice (Powell et al., 1972). Clinical trials performed in China demonstrated the efficacy of this agent against acute myeloid leukemia (AML), myelodysplastic syndrome (MDS), acute promyelocytic leukemia (APL), polycythemia vera, and central nervous system (CNS) leukemia (Kantarjian et al., 2013).

Homoharringtonine inhibits protein synthesis (Huang, 1975). More specifically, it blocks the aminoacyl-tRNA binding to free ribosomes and monosomes, but not to polyribosomes (Fresno et al., 1977). Tang et al. demonstrated that decreased expression of the antiapoptotic factor myeloid cell leukemia-1 (Mcl-1) is a key event in this antileukemic mechanism of action (Tang et al., 2006).

In 1998, a Texan biotech company developed the semisynthetic form of homoharringtonine, designated “omacetaxine mepesuccinate” (Synribo®), and provided a reliable source supply for clinical investigations by ChemGenex and the M.D. Anderson Cancer Center (Robin et al., 1999).

This preparation of homoharringtonine [Omacetaxine mepesuccinate (Synribo®)] has been granted orphan drug status in Europe and the U.S. to treat chronic myelogenous leukemia (CML). It was approved by the US FDA in October 2012 for the treatment of adult patients with CML after failure of two or more tyrosine kinase inhibitors (for a review on its clinical development, see Kantarjian et al., 2013). It is interesting to note that these approvals occurred more than 40 years after the initial discovery of this compound. Even though omacetaxine has a narrow indication in the U.S. and Europe, it has been part of standard acute myeloid leukemia (AML) therapy in China, which begs for extending its use for additional indications.

## Carfilzomib (Kyprolis®)

In 1992, Bristol-Myers Squibb scientists from Tokyo reported the structure of epoxomicin, a microbial tetrapeptide appended with an electrophilic epoxy ketone group. This compound displayed potent *in vivo* antitumor activity against murine B16 melanoma tumors. However, because the mechanism of action could not be established, its investigation was abandoned, thereby leading to the publication of the initial discovery. Eventually, BMS closed the research center in Tokyo. It was a common practice during that period for big pharmaceutical companies to close their departments of natural product chemistry.

**Scheme 3:**
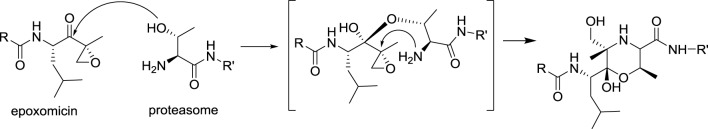
**Proposed mechanism of alkylation of the proteasome by epoxomicin**.

In 1999, the potent anticancer activity of epoxomicin attracted the attention of Craig Crews at Yale University, who designed the first synthesis of epoxomicin. In the course of this endeavor, he established the absolute configuration of the epoxide stereocenter and synthesized also a biotinylated probe, which was used to identify the proteasome as the molecular target of epoxomicin. The proteasome is a multiprotein complex that degrades unneeded or damaged proteins by proteolysis. Importantly, epoxomicin does not display any cross-inhibition with proteases, which is a major problem encountered with other anticancer proteasome inhibitors, such as bortezomib (Velcade®). The source of this selectivity was elucidated by a crystallographic approach (Groll et al., 2000). The crystal structure of the proteasome bound to epoxomicin revealed the formation of a morpholino ring between the amino terminal threonine of the proteasome and the electrophilic moiety of epoxomicin, probably through the mechanism displayed in Scheme 3.

**Figure 5:**
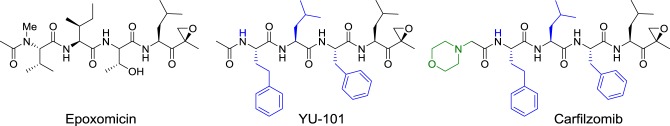
**Structures of epoxomicin, YU-101, carfilzomib**.

The specificity of epoxomicin toward proteasome prompted Crews to associate with Caltech professor Raymond Deshaies to establish a start-up company, Proteolix, dedicated to the development of a clinical candidate. During this process, they identified YU-101, which had better inhibitory activity than bortezomib (Figure 5). Addition of a morpholine moiety to YU-101 improved its solubility, thereby creating carfilzomib, which rapidly entered Phase I and II clinical trials. Importantly, the peripheral neuropathy that was observed with bortezomib did not occur with carfilzomib. In 2009, Onyx Pharmaceuticals acquired Proteolix and this compound was approved for the treatment of multiple myeloma in 2012.

## Ingenol mebutate (Picato®)

**Figure 6:**
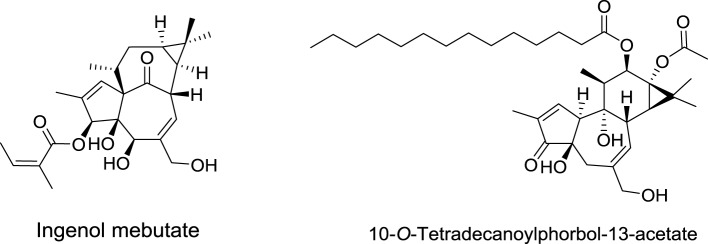
**Structures of 12-*O*-tetradecanoylphorbol-13-acetate and ingenol mebutate**.

Phorbol diesters, such as 12-*O*-tetradecanoylphorbol-13-acetate (TPA), rank among the most potent tumor promoters identified so far (Figure 6) (Nishizuka, 1984). These compounds induce tumor formation by activating protein kinase C (PKC). Interestingly, a natural compound extracted from *Euphorbia peplus* plants, Ingenol mebutate, also activates PKC but with a different pharmacological profile. Indeed, this compound induces the death of precancerous skin lesions induced by sunlight, called actinic keratosis.

The sap of *Euphorbia peplus* (known commonly as petty spurge) is commonly used as an alternative therapy for skin diseases in Australia (Weedon and Chick, 1976). In 1998, its efficacy was established for the self-treatment of skin cancers and actinic keratosis (Green and Beardmore, 1988).

Ingenol mebutate was first identified in 1980 by Evans et al. from the National Research Center in Cairo (Egypt) (Sayed et al., 1980). These authors demonstrated also that this compound is cytotoxic to cancer cells. For more than 20 years, ingenol mebutate remained poorly investigated, until 2004, when the lab of Peter Blumberg at NCI showed that it activates PKC isoforms, but with a different pharmacological profile than that of TPA. Importantly, the activation of protein kinase C delta (PKCδ) was shown to promote the production and release of inflammatory cytokines contributing to the elimination of actinic keratosis.

At the same time, Eric Raymond in Clichy (France) showed that ingenol mebutate-induced activation of PKCδ and reduced expression of PKCα lead to an activation of Ras/Raf/MAPK, an inhibition of the phosphatidylinositol 3-kinase/AKT signaling pathways, and ultimately to apoptosis of cancer cells (Benhadji et al., 2008; Serova et al., 2008; Ghoul et al., 2009).

After few years of preclinical investigations, ingenol mebutate entered clinical trials (Siller et al., 2009) and was eventually approved by FDA and EMA in 2012 for the topical treatment of actinic keratosis. This compound is produced by extraction from the petty spurge plant in low yield (1 g of pure compound/800 kg of plant). To improve the production of this molecule, Jakob Felding of LEO pharma associated with Phil Baran from Scripps Institute to develop an elegant synthesis of ingenol in only 14 steps from inexpensive (+)-3-carene (Jørgensen et al., 2013). This synthesis has been rapidly scaled-up to kilogram levels (Ritter, 2013).

## Conjugation of natural products to antibodies or folic acid to target tumors

At the end of 19th century, Paul Ehrlich already considered the conjugation of a toxin to a compound that selectively targets a disease-causing organism to generate a “magic bullet” (“*magische kugel*”) that would destroy the origin of the disease without toxicity to healthy tissues in the body (Ehrlich, 1897). About 60 years later, Mathé et al. conjugated anti-tumor antibodies to the folic acid antagonist, methotrexate (Loc et al., 1958). Although the experiments in mice were encouraging, this approach did not attract interest in the scientific community and returned to limbo for two decades, until 1975, when Ghose et al. demonstrated the efficacy of an anticancer alkylating agent, chloranbucil, conjugated to an antibody against a mouse lymphoma (Ghose et al., 1975). The advent of monoclonal antibodies the same year definitely boosted this field of research (Kohler and Milstein, 1975). Since then, almost every cytotoxic agent has been conjugated to antibodies following various strategies.

**Figure 7:**
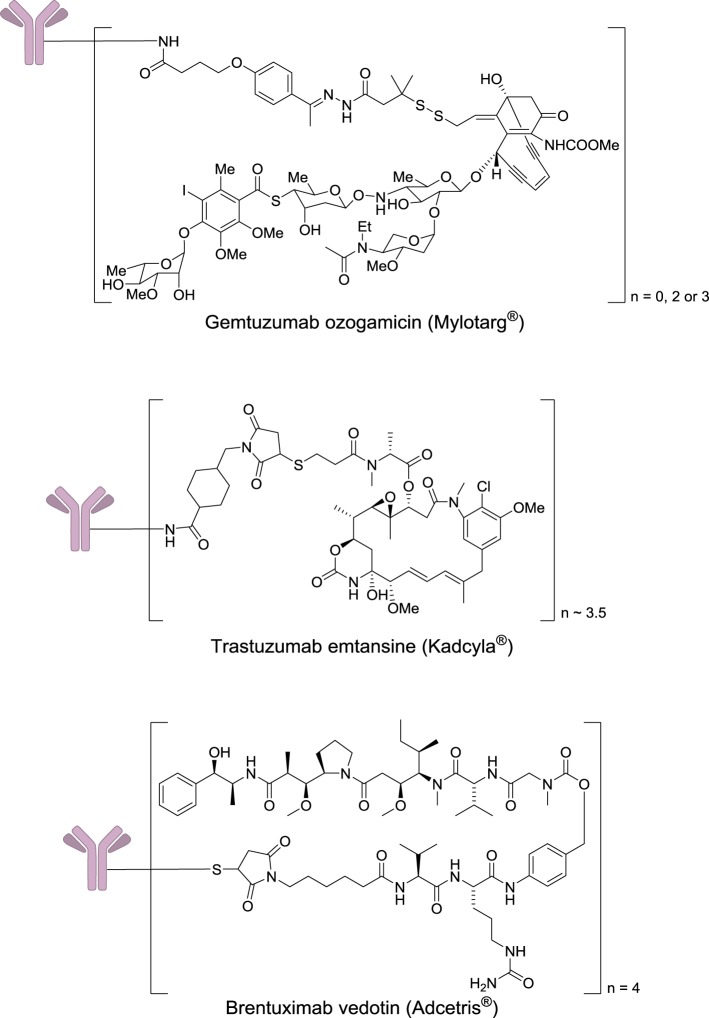
**Structures of marketed immunoconjugates**.

After two decades of endeavor, low cytotoxicity, and lack of specificity of antibodies for their targeted antigens, conjugate instability, immunogenicity, and heterogeneous product characteristics were identified as important sources of failure in the clinic (Scott et al., 2012; Ho and Chien, 2014). However, a significant step forward was made with the use of extremely highly toxic agents such as calicheamicin, maytansine, or auristatin (Figure 7). These drugs are so toxic that they cannot be used by themselves without a targeting agent.

In 2000, four decades after Mathé's pioneering work and one century after Ehrlich's dream, Wyeth received approval to commercialize Gemtuzumab ozogamicin (Mylotarg®) which results from the conjugation of a monoclonal antibody targeting CD33 with a calicheamicin derivative. This drug was used for 10 years against acute myelogenous leukemia, before being withdrawn in 2010, when it was demonstrated that it does not provide any significant benefit over conventional cancer therapies. In 2011 and 2013, two other immunoconjugates were marketed: brentuximab vedotin (Adcetris®) and trastuzumab emtansine (Kadcyla®). The first one targets the protein CD30, which is expressed in classical Hodgkin lymphoma and systemic anaplastic large cell lymphoma. This antibody is conjugated to a fully synthetic analog of the antimitotic agent dolastatin (Figure 7).

Trastuzumab emtansine results of the conjugation of a monoclonal antibody targeting the receptor HER2 (a receptor tyrosine-kinase erbB-2), which is overexpressed mainly in some forms of breast and gastric cancers to the highly cytotoxic natural product maytansine. The development of this class of agents requires a careful optimization of the monoclonal antibody, the cytotoxic payload, and the chemical linker (Ducry, 2012). The successful introduction of immunoconjugates has validated this approach to treat cancers, and currently as many as 415 antibody–drug conjugates are under clinical evaluation.

In addition to antibodies, alternative tumor-selective ligands have been conjugated to anticancer drugs. Based on observations that cells internalize vitamins, such as folate, by receptor-mediated endocytosis, Leamon, and Low from Purdue University demonstrated in 1991, that macromolecules conjugated to folic acid could be delivered into living cells (Leamon and Low, 1991). Following this seminal observation, hundreds of publications have improved upon this approach, which is currently being examined in clinical trials. The efficacy of this technology lies on the overexpression of the folate receptor in tumors, while it is quasi-absent in normal tissues. Very importantly also, folic acid retains a high affinity to its receptor when it is conjugated via its γ-carboxyl (Vlahov and Leamon, 2012).

Early attempts were limited by the release properties of the conjugates. After two decades of intensive research, some guiding rules were identified to lead compounds toward clinics:

1. anticancer agents must display a high cytotoxicity (similar to immunoconjugates);
2. enhanced hydrophilicity, to prevent passive diffusion into normal tissue;
3. an efficient cleavable linker system that releases the anticancer drug at a reliable rate once inside the targeted cell;
4. a low molecular weight, to optimize the penetration into solid tumor tissue with concomitant rapid systemic clearance.

Following these guidelines, five folic acid conjugates have reached clinical trials, including the most advanced one, vintafolide (EC145), which is currently in a phase 3 trial in women with cisplatin -resistant ovarian cancer.

**Figure 8:**
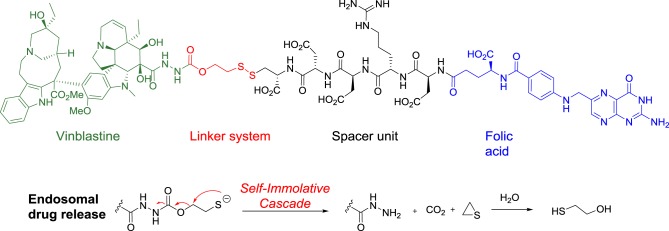
**Structure of vintafolide and mechanism of release of the payload in the endosome**.

In vintafolide, the highly cytotoxic vinblastine is connected to the folate moiety trough a self-immolative linker and a peptidic spacer (Figure 8). To provide the desired hydrophilicity to the final drug-conjugate and prevent unspecific internalization, acidic, and basic amino acids such as aspartic acid and arginine were introduced in the peptide-based unit.

The self-destructive linker system is based on a 1,2-elimination mechanism by reduction of the disulfide bond between the cysteine of the spacer and the linker, which occurs in the endosome through a not fully understood mechanism (Figure 8) (Yang et al., 2006).

## Traditional herbal remedies

In addition to purified molecules, traditional herbal remedies are slowly emerging in modern Western medicine (Basu, 2004). An injectible form of an extract of the Chinese medicinal plant *Semen coicis* called Kanglaite® (Kang-Lai-Te) has been used in China as a lipid emulsion since the end of the 90's for the treatment of non-small cell lung, liver, stomach, and breast cancers. It has been marketed also in the Russian Federation since 2003 and is the first traditional Chinese herbal remedy to enter into clinical trials in the US. As with many other traditional Chinese medicines, Kanglaite activity probably results from the combined actions of multiple pharmacologically active ingredients that have not been yet identified (Xu, 2011). Over the last decade, other botanical drugs have entered clinical trials in the West to treat cancers or other ailments.

## Nanoparticle delivery of anticancer drugs

Tumor growth requires angiogenesis, i.e., the formation of new blood vessels. In contrast to normal angiogenesis, newly formed vessels in tumors display many structural and functional defects, which permit the leakage of macromolecules. This feature is referred to as the “enhanced permeability and retention (EPR) effect.” Recent advances in the application of nanotechnology to medicine enabled the approval of five nanoparticle chemotherapeutics for cancer (Wang et al., 2012). Four liposomal formulations have been approved for clinical use in oncology: pegylated liposomal doxorubicin (DOXIL®, Caelyx®), nonpegylated liposomal doxorubicin (Myocet®), and liposomal cytarabine (DepoCyte®) (Hofheinz et al., 2005). Nab-paclitaxel (Abraxane®) is an albumin bound approved for the treatment of breast cancer and is undergoing clinical trials for other clinical indications. And finally, Genexol-PM is a polymeric micelle formulation of paclitaxel composed of block copolymers of PEG and poly-(D,L-lactic acid) (Kim et al., 2004).

Although nanomedicine is a new discipline, its translation into clinics has been rapid. A novel generation of nanoparticle chemotherapeutics is under development and expected to greatly improve cancer treatments. These new formulations may also offer novel opportunities for established anticancer drugs (Wang et al., 2012).

## Missed opportunities and how to rescue them

In 2010, Bristol-Myers Squibb stopped the phase III clinical trial of Tanespimycin, an inhibitor of heat shock protein 90, for the treatment of multiple myeloma, probably because of the expiration of the patent in 2014. In addition to drug developments that were terminated because of the shortness of patent life, there are many interesting drugs that did not reach clinical trial or that failed in clinical trial because the conceptual tools to correctly perform these assays were not available at that time. Indeed, “there are no bad anticancer agents, only bad clinical trial designs” as stated by Von Hoff (1998).

Flavaglines, such as rocaglamide, represent a striking example of natural products that are enjoying reinvigorated investigation after their original discovery by King et al. from the National Defense Medical Center of Taiwan (King et al., 1985). The recent identification of their molecular targets, the scaffold proteins prohibitins and the initiation factor of translation eIF4A, coupled with a description in *Science* about the origin of their selective cytotoxicity in cancer cells should promote further investigations to unveil their therapeutic usefulness (Basmadjian et al., 2013; Santagata et al., 2013). However, clinical trials with these compounds are unlikely unless some structurally original and patentable analogs are identified. Indeed, clinical trials of non-patentable compounds are still scarce (Roin, 2009; Cvek, 2012). For instance, a non-profit company, the Institute for OneWorld Health, developed in 2007 paromomycin, which is not patentable, as an effective treatment for visceral leishmaniasis. This was accomplished with financial support from the Bill and Melinda Gates Foundation, the Special Program for Research and Training in Tropical Diseases of the United Nations Development Program, the World Bank, and the World Health Organization (Sundar et al., 2007). GlobalCures is another example of a non-profit medical research organization, which aims to develop novel and cost-effective treatments for cancers (Cvek, 2012). State agencies, such as the National Center for Advancing Translational Sciences are also deeply involved in the development of non-profitable drugs. Only a radical change in public or international policy could support the further development of clinically useful compounds that are currently fated to be traded as generics.

## Biotechnology-based generation of novel natural products

**Scheme 4:**
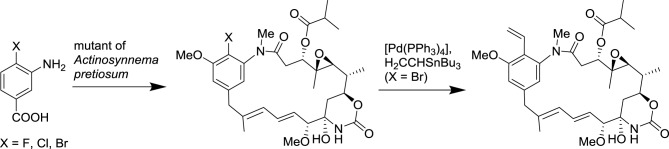
**Combination of biotechnology and organic synthesis for the synthesis of ansamitocin derivatives (Taft et al., 2008)**.

Since the seminal synthesis of aspirin by Gerhardt (1853), all the natural product derivatives were prepared by total synthesis or semi-synthesis. Alternate approaches are currently emerging based on the progress in the deciphering of biosynthetic pathways and advances in biotechnologies. Currently, only a tiny fraction of microbes can be cultured with conventional approaches, yet uncultivated microorganisms represent an attractive source of novel natural products. It is now possible to isolate large fragments of microbial DNA directly from environmental samples and to express them in an easily cultured microorganism. This approach provides access to secondary metabolites that were originally produced by inaccessible microorganisms. Additionally, the manipulation of these biosynthetic pathways can lead to novel natural product derivatives. Metabolic engineering and synthetic biology are poised to revolutionize conventional chemical and pharmaceutical manufacturing in the coming decade (Yadav et al., 2012). Recently, methods and concepts of organic synthesis have begun to be integrated to synthetic biology to generate novel natural product derivatives. Such approaches that merge biotechnology with organic synthesis are rapidly blooming and are expected to efficiently generate novel natural product analogs in the near future (Goss et al., 2012; Kirschning and Hahn, 2012). A representative example of such an approach has been the use of an *Actinosynnema pretiosum* mutant that accepts 3-amino-4-bromobenzoic acid as a substrate to prepare pharmacologically active ansamitocin derivatives, which can then be transformed by classical organic reactions (Scheme 4; Taft et al., 2008).

## Conclusion

The success of glivec and herceptin in the 90's announced the obsolescence of natural products in therapeutics. A decade later, many cancer patients continue to die and pharmaceutical companies have reconsidered their position on the potential of natural products in oncology. Indeed, for too many solid tumors of advanced grades, the only therapeutic options remain exclusively palliative. There is therefore an urgent need to develop original medicines.

Some of newly developed agents induce a strong cytotoxicity targeting conventional targets, DNA (for trabectedin) or microtubules (for ixabepilone, vinflunine, or eribulin), while other target specific biochemical events such as steroid biosynthesis (abiraterone acetate), histone remodeling (for romidepsin), protein translation (homoharringtonine), or degradation (carfilzomib). The case of rapamycin derivatives is atypical. These drugs are not cytotoxic, but can be considered as targeted therapy agents due to their inhibition of mTOR signaling.

In contrast with targeted therapeutics, which are designed for a specific type of cancer, the development of natural products is often more erratic and heavily relies on the skill of pharmacologists to unravel their mechanism of action and clinicians to identify the optimal indication in the clinic.

Over the last 15 years, natural products have been rehabilitated by pharmaceutical companies, even though some complementary approaches, such as molecular modeling based drug design are gaining in momentum. This latter methodology, which was pioneered by 2013 Nobel laureates, has successfully led to innovative medicines. When it is possible to predict the 3D structure of proteins, then it will probably overshadow other methods for identifying drug candidates. Until then, natural products should continue to play a major role in drug discovery, especially in the treatment of cancers and infectious diseases.

### Conflict of interest statement

The authors declare that the research was conducted in the absence of any commercial or financial relationships that could be construed as a potential conflict of interest.
